# Relapsing Fever Infection Manifesting as Aseptic Meningitis, Texas, USA

**DOI:** 10.3201/eid2710.210189

**Published:** 2021-10

**Authors:** Lisa Ellis, Michael W. Curtis, Sarah M. Gunter, Job E. Lopez

**Affiliations:** Austin Infectious Disease Consultants, Austin, Texas, USA (L. Ellis);; Baylor College of Medicine, Houston, Texas (M.W. Curtis, S.M. Gunter, J.E. Lopez)

**Keywords:** aseptic meningitis, spirochetes, neuroborreliosis, Borrelia turicatae, relapsing fever, tickborne, bacteria, vector-borne infections, Texas, United States

## Abstract

Tickborne relapsing fever spirochetes are an overlooked cause of disease around the globe. We report a case of tickborne relapsing fever in a patient in Texas, USA, who had a single febrile episode and gastrointestinal and neurologic symptoms. Immunoblot analysis using recombinant *Borrelia* immunogenic protein A implicated *Borrelia turicatae* as the causative agent.

## Abstract 2

Neuroborreliosis initially misdiagnosed as Lyme disease was discovered to be caused by the relapsing fever spirochete *Borrelia turicatae*.

Tickborne relapsing fever (TBRF) spirochetes are globally neglected pathogens. *Borrelia turicatae* is found in the southwestern and eastern United States into Latin America (*1*), and high-risk populations include military personnel, outdoor enthusiasts, and impoverished undocumented immigrants (*2*–*4*). However, evidence indicates the presence of endemic foci of *B. turicatae* in metropolitan cities of Texas, USA (*4*,*5*).

TBRF is often misdiagnosed because of the nonspecific manifestations of the disease. More than 90% of patients experience recurrent febrile episodes, rigors, headache, and myalgia (*6*). Previous work suggests that *B. turicatae* is similar to Old World species, manifesting with neurologic complications (*7*). However, these diagnoses were made on the basis of a priori assumptions, and the causative agents were never confirmed.

We report a case of neuroborreliosis in Austin, Texas, USA, that was initially suspected to be Lyme disease (LD). A retrospective serologic analysis was performed using the diagnostic antigen, *Borrelia* immunogenic protein A (BipA). This antigen is absent from LD-causing spirochetes and might be a species-specific antigen for North American TBRF *Borrelia* (*8*,*9*). 

## The Study

**Figure 1:**
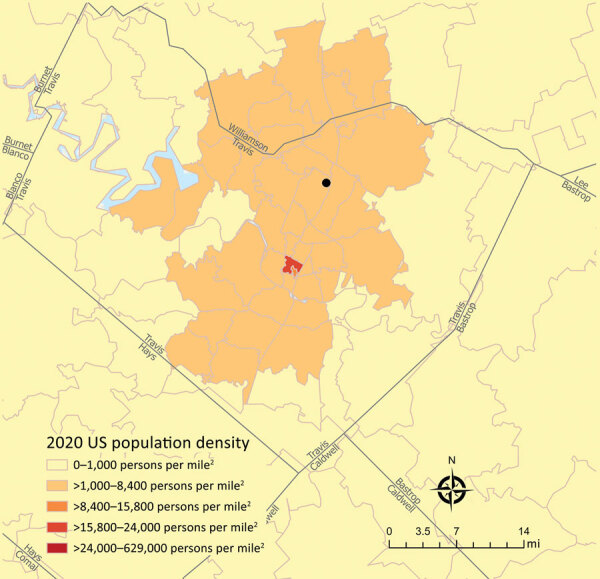
Suspected *Borrelia* exposure site within city limits for a patient in Austin, Texas, USA. The patient’s suspected exposure location (black circle, Walnut Creek Metropolitan Park) was overlayed on a population density by ZIP code map. County boundaries are displayed as gray lines. Population density data was sourced from Esri's U.S. Updated Demographic (2020/2025) Data (https://www.esri.com).

The patient was a previously healthy 30-year-old man residing in Austin near a creek greenbelt that he frequented (Figure 1); he had no recent travel outside the city. On March 5, 2020, he experienced acute dizziness, headache, myalgia, vomiting, chills, and fever of 37.8°C (reference 36.1°C–37.2°C). Symptoms were attributed to a foodborne illness, and he improved after several days. However, he continued to experience dizziness, headache, fatigue, myalgia, and intermittent severe night sweats, with no report of further fever.

Within 2 weeks, he had Bell’s palsy on his left side, and the primary care physician ordered a blood analysis. Results for complete blood count, electrolytes, blood urea nitrogen, creatinine, and liver enzymes were unremarkable. Erythrocyte sedimentation rate was 62 mm/h (reference <15 mm/h), and C-reactive protein was 97.5 mg/L (reference <8.0 mg/L). Valacyclovir (1 g orally 3×/d for 7 d) and prednisone (20 mg orally 2×/d for 5 d) were prescribed, with partial improvement. Subsequent blood testing showed the erythrocyte sedimentation rate declined to 41 mm/h, and C-reactive protein declined to 43.2 mg/L.

Two weeks later, he had Bell’s palsy on his right side, blurred vision, tinnitus, and cervical lymph node enlargement. Dizziness, headache, and fatigue continued. Results of complete blood count and metabolic panel were unremarkable. The patient underwent magnetic resonance imaging of the brain with contrast, which revealed faint nonspecific enhancement in the right internal auditory canal. High-resolution imaging of the 7th and 8th cranial nerves was not performed. 

The patient’s wife reported removing ticks from herself and a pet 4 weeks before the patient’s illness began, and LD was suspected. A 2-tiered antibody test was performed. The enzyme immunoassay result was 2.43 (>1.09 considered positive). The LD IgM immunoblot was positive for the 23 kDa and 39 kDa bands, but the IgG immunoblot was negative.

The patient was referred to an infectious disease specialist who suspected TBRF and ordered a lumbar puncture. Clear, colorless cerebrospinal fluid (CSF) was recovered, and the analysis revealed 124 leukocytes/UL (reference <5 leukocytes/UL) with 85% lymphocytes, 10% monocytes, 5% large mononuclear cells, and 0% erythrocytes/UL. CSF protein was 103 mg/dL (reference 15.0–45.0 mg/dL), and glucose was 52 mg/dL (reference 40–70 mg/dL). CSF was analyzed by the Associated Regional and University Pathologists laboratory for LD and TBRF spirochete DNA, the Venereal Disease Research Laboratory test for neurosyphillis, and the Biofire Filmarray Meningitis/Encephalitis (bioMérieux, https://www.biomerieux-usa.com) panel that detects 6 bacterial and 7 viral pathogens. All test results were negative.

Given the patient’s clinical history, intravenous ceftriaxone was administered (2 g/d for 14 d), and he showed considerable improvement within 4 days of treatment. Upon completion of antibiotics, all symptoms were resolved except for minimal lower right facial weakness. Deidentified serum samples and CSF collected 5 weeks after the onset of illness were sent to Baylor College of Medicine (Houston, TX, USA) for additional testing.

No spirochetes were recovered from the CSF nor was DNA detected; therefore, we performed serologic tests using recombinant BipA (rBipA) (*8*,*9*). We generated expression constructs for *B. turicatae*, *B. parkeri*, and *B. hermsii* rBipA by using GenScript (GenScript, https://www.genscript.com) in the pET19b vector. We purified recombinant proteins and performed immunoblotting and ELISA, as previously described (*8*). For immunoblots, we used protein lysates from *B. turicatae* 91E135, *B. hermsii* DAH, and *B. parkeri* SLO. We probed immunoblots with the patient’s serum sample and CSF diluted 1:200. Only the serum sample was diluted 2-fold from 1:200 to 1:256,000 for the ELISA because the CSF was depleted in prior assays. The secondary antibody was goat anti-human IgA, IgG, and IgM (Millipore, https://www.emdmillipore.com). We repeated serologic assays twice.

**Figure 2:**
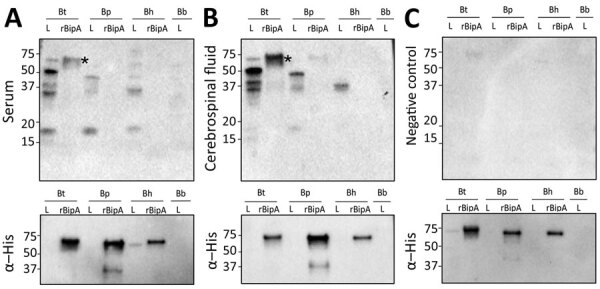
Immunoblots assessing antibody responses to *Borrelia* protein lysates and rBipA in samples from a patient in Texas, USA, and a control sample. A, B) Serum (A, upper panel) and cerebrospinal fluid (B, upper panel) samples were used to detect reactivity to *Borrelia* protein lysates and to rBipA from each species of tickborne relapsing fever spirochete. C) Negative human serum sample (upper panel) and immunoblots (bottom panel) that were reprobed with a monoclonal antibody for the histidine residues fused on the N terminus of each recombinant protein. Asterisks (*) indicates rBipA, which is ≈65 kDa. Molecular masses in kDa are indicated on the left of each immunoblot. Bb, *Borreliela* (*Borrelia*) *burgdorferi*; Bh, *B. hermsii*; BipA, *Borrelia* immunogenic protein A; Bp, *B. parkeri*; Bt, *B. turicatae*; rBipA, recombinant BipA.

Serologic assays indicated likely exposure to *B. turicatae.* Strong responses were detected with the serum sample and CSF to *B. turicatae* protein lysates and rBipA (Figure 2, panels A and B upper). Antibodies in the patient’s serum and CSF cross-reacted with protein lysates from *B. parkeri* and *B. hermsii*, but reactivity to rBipA from these species was undetectable (Figure 2, panels A, B, top images). In addition, reactivity to *B. burgdorferi* protein lysates was undetectable (Figure 2, panels A, B, top images). A negative control serum sample from a subject without history of TBRF failed to detect proteins (Figure 2, panel C, top image). Reprobing immunoblots with a monoclonal antibody for the histidine residues fused to rBipA demonstrated that protein was electrophoresed and transferred to membranes (Figure 2, bottom images). ELISA further indicated infection attributable to *B. turicatae* with antibody titers to *B. turicatae* rBipA between 1:400 to 1:800, and responses to *B. parkeri* and *B. hermsii* rBipA were undetectable.

## Conclusions

This study reports a case of neurologic TBRF likely caused by *B. turicatae*. The hallmark of TBRF is recurrent febrile episodes (*6*), but this patient had a single febrile episode, nausea, and predominantly neurologic symptoms. *B. turicatae* has been suspected to cause neurologic symptoms including facial paralysis, vertigo, hearing loss, delirium, and hallucinations (*10*). However, past diagnoses were attributed solely on the basis of the geographic range of the probable pathogen and were not empirically confirmed.

This study demonstrated that rBipA could aid in the identification of the TBRF species causing infection. It was unlikely that the patient was exposed to *B. hermsii* and *B. parkeri* because of his travel history, but we used this opportunity to assess serologic crossreactivity to rBipA from these 2 species. Similar to prior work with *B. turicatae*–infected laboratory animals (*8*), we detected no crossreactive patient antibodies to *B. hermsii* rBipA. This finding was expected given that the proteins share ≈35% amino acid identity (*11*). Of note, rBipA could differentiate between infections caused by *B. parkeri* and *B. turicatae,* which share ≈75% amino acid identity (*11*).

In summary, *B. turicatae* is often misdiagnosed, and healthcare providers should understand the pathogen’s circulation (*2*,*5*,*11*). Endemic foci have been identified in Florida, USA, and within and around the 4 largest cities of Texas (Austin, San Antonio, Dallas, and Houston) (*1*–*3*,*5*,*7*). With urban expansion and the incorporation of greenbelts into metropolitan areas, *B. turicatae* should be considered in cases of fever with neurologic symptoms when the Lyme antibody test is positive but prevalence of LD is not epidemiologically supported.
